# Infant and child feeding index reflects feeding practices, nutritional status of urban slum children

**DOI:** 10.1186/s12887-014-0290-7

**Published:** 2014-11-30

**Authors:** Neha Lohia, Shobha A Udipi

**Affiliations:** Department of Food Science and Nutrition, SNDT Women’s University, Sir Vithaldas Vidyavihar, Juhu, Mumbai, 400 049 India

**Keywords:** Infant and child feeding index, Dietary diversity, Complementary feeding practices, Nutritional status, Urban slums, India

## Abstract

**Background:**

Infant and child feeding index (ICFI) an age-specific index, can be used to assess child feeding practices. We used the ICFI to assess feeding practices for urban slum children and the association between ICFI and child nutritional status.

**Methods:**

446 children aged 6 to 24 months from urban slums of Mumbai, India were studied. We used the 24-hour diet recall to study dietary diversity and a food frequency questionnaire for consumption of food groups during the preceding week. ICFI was computed using five components, namely, breastfeeding, use of bottle, dietary diversity score (DDS), food group frequency score (FGFS) and feeding frequency scores (FFS). Weight, height and Mid-Upper Arm Circumference (MUAC) were measured, and z scores were calculated. Association between ICFI scores and nutritional status was examined.

**Results:**

The mean total ICFI score for all was 5.9 ± 1.9. Among the five components, FGFS and FFS differed between children <12 months of age and >12 months and by breast feeding status. In contrast, there were no differences vis-à-vis dietary diversity scores (DDS), breast feeding, and use of bottle. Non-breastfed children had significantly higher DDS scores than did breastfed children. The mean feeding frequency score (FFS) for children <12 months of age was slightly but not significantly lower than scores for children >12 months of age. Mother’s age and child’s age were significant determinants of ICFI. Multivariate analysis indicated that ICFI was significantly associated with Length-for-Age z scores (LAZ) and BMI-for-Age z scores (BAZ). Sensitivity of ICFI was lower than its specificity.

**Conclusions:**

The results of the present study confirmed that the ICFI can be used to collect information on key components of young child feeding practices and be incorporated into public-health programmes. Further, it could be used to determine the influence of complementary feeding practices on nutritional status of children.

## Background

Globally, more than one-third of child deaths occur due to undernutrition, which is more prevalent in low- and lower-middle-income countries [1,2]. In India, the third National and Family Health Survey [3] indicated that 46% of children under three years were underweight, 38% were stunted and 19% were wasted. Infants and young children upto two years of age are considered to be the most vulnerable because of their higher requirements of energy- and nutrient- dense foods to support their growth and physical and mental development [4]. Hence, infant and young child feeding practices (IYCF) during this period play a critical role. Faulty breastfeeding and poor complementary feeding can lead to undernutrition [5-7].

In the Indian context, most of the reports in the literature have focused on specific feeding behaviours such as breastfeeding, age at introduction of complementary foods [3,8-10]. However, all these studies have not captured the multidimensionality of feeding practices including dietary diversity and have not examined their influence on child nutritional status.

IYCF practices are multidimensional and age-specific. Ruel and Menon developed a composite feeding index to identify nutritionally vulnerable children [11]. This index is based on an age-specific scoring system that gives points for positive practices such as breastfeeding, avoiding use of bottle for feeding, meal frequency and dietary diversity. Efforts to measure and quantify IYCF practices using ICFI and to determine its association with nutritional status have been reported by various investigators in different countries [7,12-16]. Many investigators [5-7,13,15,16] have shown a positive association between ICFI and nutritional status. While none of the studies indicated a significant association between all the three indicators of nutritional status i.e. weight-for-age (WAZ), weight-for-length (WLZ) and length-for-age (LAZ), most investigators have found an association with LAZ [6,7,13,15,16]. In India, only two groups of investigators have used a scoring system for complementary feeding practices to determine the association with nutritional status of young children [5,6]. Therefore we used the index developed by Ruel and Menon [11] to assess feeding behaviours and to identify which of the ICFI components may require attention in nutrition education interventions in Indian slums. The data reported herein is the baseline data which was part of a longitudinal intervention on feeding practices of young children aged 6 to 24 months. Feeding practices were examined using the ICFI. The association between ICFI and child nutritional status was studied. We also calculated the specificity and sensitivity of the ICFI in order to determine whether it can serve to identify undernourished children.

## Methods

### Study site and sample selection

**Figure 1:**
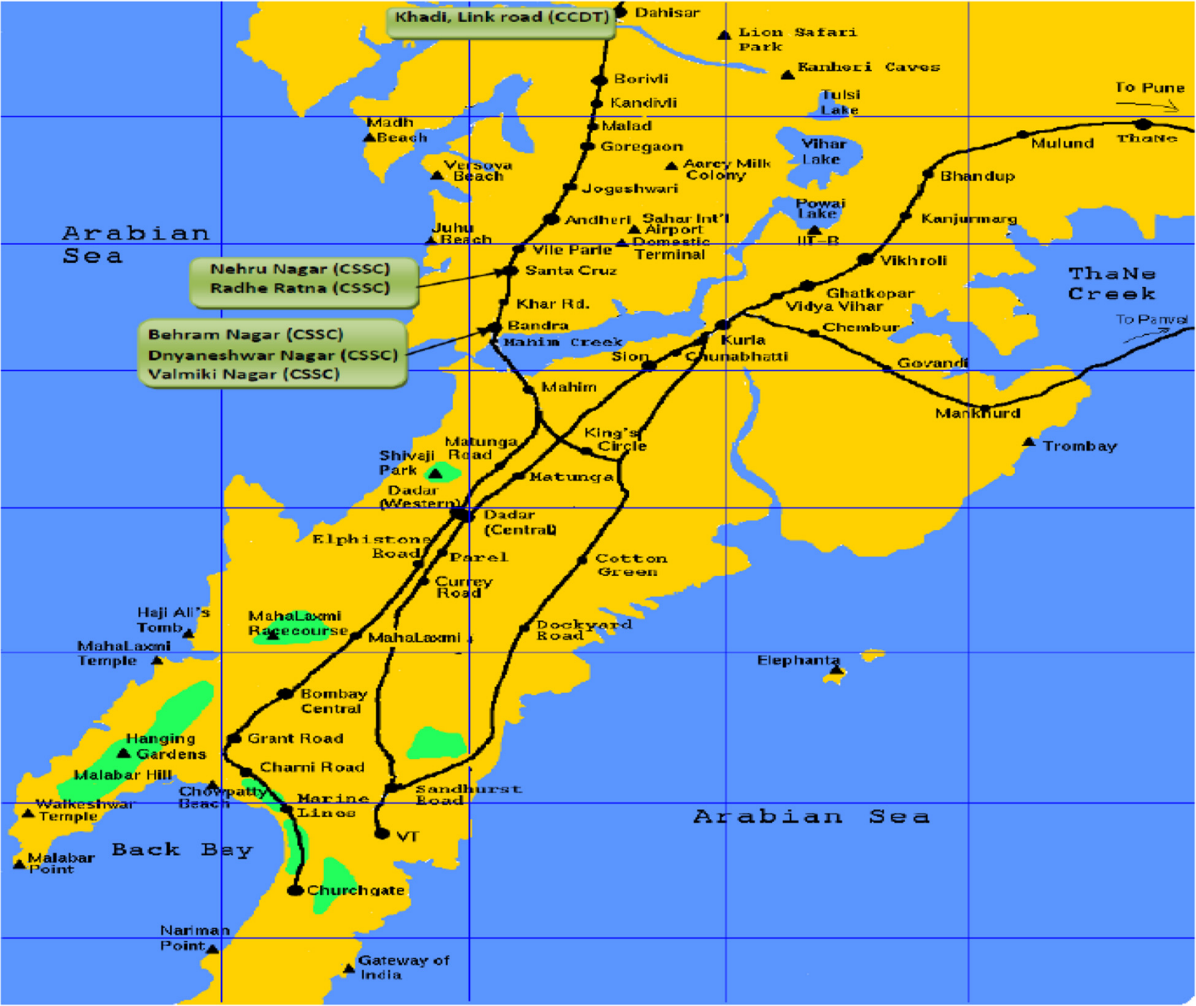
**Locations of the slums selected for the study.**

This study was conducted in six slums adopted by two non-governmental organizations- Committed Communities Development Trust (CCDT) and Centre for the Study of Social Change (CSSC). The slums are located in three western suburbs of Mumbai city indicated in the map (Figure 1). Each slum had a population of approximately 1000 families. A little less than half of the families (47.9%) had houses made of asbestos/tin. One-fourth of the families (26.9%) lived in houses constructed from brick/stone wall and RCC roof and the remaining families were either residing in transit camp (12.6%) or in a semi – pucca house with brick walls and tin roofs (12.6%). Almost three-fourths of the families were Hindu (75.8%) and the remaining 24.2% were Muslim. The mean number of family members was 6.1 ± 3.1. Mean total family income was Rs. 8067 ± 6836 and mean per capita income was Rs. 1436 ± 974/ month. More than two – thirds of mothers (69.3%) were literate.

The data reported in this study is part of the baseline data collected during a longitudinal study. The main study aimed to compare the impact of nutrition education with and without ready – to – use food supplement on growth, feeding and nutrient intakes of young children through improvement of complementary feeding practices of mothers.

Using 90% power, the number of mother – child pairs required was 150 in each intervention group. Hence, it was decided to recruit at least 200 mother – child pairs in each group, taking into account possible dropouts.

All mothers in the selected slums who had children aged 6 to 24 months were included in the study based on the following criteria: willingness of mother to participate in the study, child should not be exclusively breastfed and it should not have any congenital anomalies or disease or any kind of food allergy. Four hundred and forty six mother-child pairs aged 6 to 24 months who met the inclusion criteria were included in the study.

### Data collection

Information about mother/caregiver education, age, family income and about child characteristics such as age, ordinal position and gender was obtained by interviewing mothers. Anthropometric measurements of children were taken thrice and the average of three readings was calculated. Weight was measured using a Salter scale calibrated to the nearest 100 g with a maximum capacity of 25 kg. Length was measured using an infantometer to the nearest 0.1 cm. Mid-upper arm circumference (MUAC) was measured to the nearest 0.1 cm using a flexible, non-stretchable measuring tape standardized against a stadiometer.

Mother’s height and weight were measured twice. Weight was measured using an Equinox digital weighing balance accurate to 100 g and height was measured using a non-stretchable measuring tape standardized against a stadiometer. BMI was calculated.

### Infant and Child Feeding Index (ICFI)

The ICFI was constructed as described by Arimond and Ruel and Moursi et al. [14,17]. The variables and scoring system used are shown in Table 1 [14]. Mothers were interviewed about infant feeding practices and consumption of food groups during the week preceding the survey. Dietary diversity scores were calculated with the use of a 24-hr dietary recall. Food frequency questionnaire (FFQ) was used to assess food group frequency score (FGFS).

**Table 1:** **Variables and scoring system used to construct the infant & child feeding index**

**Variables**	**6 – 8 Mo**	**9 – 11 Mo**	**12 – 24 Mo**
Breastfeeding	Yes – 2	Yes – 2	Yes – 1
	No – 0	No – 0	No – 0
Bottle feeding	Yes – 0	Yes – 0	Yes – 0
	No – 1	No – 1	No – 1
Dietary diversity score^1^ (24-h recall)	0– 1 food group – 0	0– 2 food group – 0	0– 2 food group – 0
	2 food groups – 1	3 food groups – 1	3 food groups – 1
	≥3 food groups – 2	≥4 food groups – 2	≥4 food groups – 2
Food group frequency score^1,2^ (past 7d – food frequency questionnaire)	0 – 2 food groups – 0	0 – 3 food groups – 0	0 – 3 food groups – 0
	3 – 4 food groups – 1	4 food groups – 1	4 food groups – 1
	≥5 food groups – 2	≥5 food groups – 2	≥5 food groups – 2
Feeding frequency	0 – 1 times – 0	0 – 2 times – 0	0 – 2 times – 0
	2 times – 1	3 times – 1	3 times – 1
	≥3 times – 2	≥4 times – 2	4 times – 2
			≥5 times – 3

^1^Food groups: grains/roots/tubers; legumes/nuts; dairy; flesh foods; eggs; vitamin A rich fruits & vegetables; other fruits & vegetables.

^2^Each food group scored 0 if not consumed during the previous week, +1 if consumed on 1 to 3 days and +2 if consumed on 4 days/more. These scores were summed to give a possible range of 0 – 14 & then new food group frequency scores were assigned as described [14].

For each child, one – day 24-hour semi-quantitative recall was taken using the multiple pass method. The FFQ consisted of 50 items belonging to 9 food groups – i)Tea/milk, ii) Cereal preparations, iii) Pulse preparations, iv) Cereal and pulse combinations, v) Vegetables, vi) Fruits, vii) Biscuits, viii) Non vegetarian items and ix) Other foods such as sago, sugar, ice-cream, commercial weaning foods.

Dietary diversity score was calculated by adding the number of food groups [14] consumed on the previous day of the survey. The food group frequency score, was assessed separately by using food frequency questionnaire; each food group was scored 0 if not consumed during previous week, +1 if consumed on 1–3 days and +2 if consumed on ≥4 days. The distribution of feeding frequency scores was assessed for each age range, a score of +1 was given to children who met the recommendation of feeding 2 times/day for 6–8 months, 3 times/day for 9 months or more, and a score of +2 was given when children who met recommendation of feeding ≥3 times/day for 6–8 months and ≥4 times/day for >9 months. Older children, who were fed for ≥5 times /day, were assigned a score of +3. The ICFI score was calculated by adding up the scores obtained, giving a range of 0–9. Further, the ICFI scores were categorised as: *low* = a score of 0–5, *medium* = 6-7 and *high* = 8-9 [14].

### Ethics

The study protocol was reviewed and approved by the Intersystem Biomedical Ethics Committee. Informed written consent was taken from all the mothers who participated in the study.

### Data analysis

Data was analyzed using the SPSS software (version 20.0). Standard deviation/ Z scores were calculated using the WHO Anthro software (2009) for weight for age (WAZ), length for age (LAZ), weight for length (WLZ), MUAC for age (MUACZ) and body mass index (BMI) for age (BAZ) values. Also children were classified into various grades of nutritional status based on the WHO criteria [18]. For examining association of ICFI scores with age, children were divided into four age groups 6 – 8.99, 9 – 11.99, 12 – 17.99 and 18 – 24 months.

Distribution of quantitative variables was analysed using ANOVA and χ^2^ test. For multivariate analysis, multiple linear regression analysis was done for each of the dependent variables, including WLZ, WAZ, LAZ, BAZ and MUACZ after adjusting for maternal and child characteristics that were considered to be confounders. A final model was developed for each of the nutritional parameters that included ICFI, age of the mother, education of mother, BMI of mother, family income, age of child, gender of child and ordinal position. All the test variables were considered significant for a p value <0.05. The sensitivity and specificity of the ICFI were measured to examine the accuracy of ICFI for correctly identifying the children as wasted, stunted and underweight. In this study, sensitivity was defined as ability of ICFI to correctly identify the children as wasted (WAZ < −2SD; MUACZ < −2SD), stunted (LAZ < −2SD) and underweight (WLZ < −2SD, BAZ < −2SD) when the ICFI score was ≤5 and specificity was defined as ability of ICFI to correctly identify children as normal (WLZ, WAZ, LAZ, BAZ and MUACZ > −2SD) when the ICFI score was 6 or more.

## Results

### Sample characteristics

The mean age of mothers was 25.3 ± 3.9 years, and the mean BMI was 21.1 ± 4.2 kg/m^2^. More than half of the mothers (56.7%) had normal BMI, with one-fourth of them being underweight (27.4%). One-third of mothers (30.7%) in the study were illiterate, two-thirds of the mothers had completed primary or secondary schooling (61.0%) and only 8.3% mothers had completed higher secondary or graduation. The mean age of the children was 12.7 ± 4.6 months, with 49.1% being male and 50.9% being female. The mean family income was INR 8066.70 ± 6836.4 with the mean per capita income being INR 1435.7 ± 974.3.

### Nutritional status

A little more than one-fourth of the children (26.7%) were underweight (WLZ < −2 SD and BAZ < −2SD), half of the children (51.3%) were stunted (LAZ < −2SD) and 41.7% were wasted (WAZ < −2SD). Only 13.5% children had a MUACZ score < −2SD. The mean WLZ score of children >12 months of age was significantly better compared to children <12 months of age (F value = 6.275, p = 0.013). The mean LAZ scores of children above 12 months of age were lower compared to children below 12 months of age (F = 88.039, p = 0.000). With respect to WAZ and BAZ, the mean z scores were significantly better among children >12 months than the younger age children (WAZ: F value = 14.885, p = 0.000; BAZ: F value = 23.087, p = 0.000). In contrast, the mean MUACZ score was better among children <12 months of age compared to older children (F value = 10.410, p = 0.001).

The association between mean z scores and gender was examined. There was no significant association between gender and mean WLZ, WAZ, BAZ and MUACZ scores. However, a significant association was found between mean LAZ scores and gender. The mean z- score for males was −2.48 ± 2.6, that was lower than the mean score for females (−1.96 ± 2.5; F value = 4.692, p = 0.031). There was no significant association between breastfeeding status and nutritional status.

### ICFI Score

The mean total ICFI score of all the subjects in the study was 5.9 ± 1.9.The mean score was calculated by gender of the child, breastfeeding status and age categories (Table 2). Breastfeeding was found to be an important influence, as breast fed children had significantly higher mean ICFI scores than non breast fed children (F value = 8.111, p = 0.005) especially among the <12 months age group. No significant gender difference was observed in the scores (F value = 0.165, p = 0.685). Breastfed children aged <12 months had higher ICFI score but children above 9 months of age had slightly but not significantly lower scores than the children below 9 months of age (F value = 0.574,p = 0.634). There was significant interaction between breast feeding status and age category (F value = 4.131, p = 0.007) but not between sex and age category (F value = 0.401, p = 0.753). Also, the interaction between all three variables was not statistically significant (F value = 0.342, p = 0.795).

**Table 2:** **Mean total ICFI Scores by gender and age categories**

**Age categories**	**Breastfed**			**Non–breastfed**			**All children**		
	**(n = 381)**			**(n = 65)**			**(n = 446)**		
	**Male**	**Female**	**Total**	**Male**	**Female**	**Total**	**Male**	**Female**	**Total**
	**(n = 95)**	**(n = 95)**	**(n = 190)**	**(n = 124)**	**(n = 132)**	**(n = 256)**	**(n = 219)**	**(n = 227)**	**(n = 446)**
6 – 8.99 mos (n = 100)	7.1 ± 1.7	6.0 ± 1.8	6.5 ± 1.8	5.3 ± 0.9	5.0	5.2 ± 0.8	6.9 ± 1.7	6.0 ± 1.7	6.5 ± 1.8
9 – 11.99 mos (n = 90)	6.3 ± 1.7	5.8 ± 1.8	6.1 ± 1.8	4.3 ± 1.5	4.6 ± 1.7	4.4 ± 1.5	6.2 ± 1.8	5.6 ± 1.8	5.9 ± 1.8
12 – 17.99 mos (n = 196)	5.4 ± 2.2	5.6 ± 2.0	5.5 ± 2.1	5.6 ± 0.9	6.1 ± 1.9	5.9 ± 1.6	5.5 ± 2.0	5.7 ± 2.0	5.6 ± 2.0
18 – 24 mos (n = 60)	6.1 ± 1.9	6.4 ± 2.0	6.3 ± 1.9	5.1 ± 1.9	4.7 ± 1.2	4.9 ± 1.7	5.8 ± 1.9	5.8 ± 1.9	5.9 ± 1.9
All children	6.1 ± 2.0	5.8 ± 1.9	5.9 ± 1.9	5.3 ± 1.4	5.6 ± 1.8	5.4 ± 1.6	6.0 ± 1.9	5.8 ± 1.9	5.9 ± 1.9

Mean values ± standard deviation.

### Components of ICFI

Mean scores for each component were compared between age groups and by sex (Table 3). Mean scores for each component did not differ significantly by gender except for the scores for breastfeeding. When age categories were compared, the older children had significantly lower scores for BF. Mean scores for bottle feeding did not differ with age.

**Table 3:** **Mean scores for components of icfi by gender and by age categories**

**ICFI components**	**Male (n = 219)**	**Female (n = 227)**	**All children (n = 446)**	**F value, p (by gender)**
**Breastfeeding**				
6 – 8.99 mos	1.9 ± 0.5	2.0 ± 0.3	1.9 ± 0.4	15.174,0.000
9 – 11.99 mos	1.9 ± 0.5	1.8 ± 0.6	1.8 ± 0.6	
12 – 17.99 mos	0.9 ± 0.4	0.8 ± 0.4	0.8 ± 0.4	
18 – 24 mos	0.7 ± 0.5	0.8 ± 0.4	0.7 ± 0.4	
**F value, p (by age category)**	161.758,0.000			
**Bottle-feeding**				
6 – 8.99 mos	1.0 ± 0.2	1.0 ± 0.1	1.0 ± 0.2	1.388,0.239
9 – 11.99 mos	1.0 ± 0.2	1.0 ± 0.2	1.0 ± 0.2	
12 – 17.99 mos	1.0 ± 0.1	1.0 ± 0.1	1.0 ± 0.1	
18 – 24 mos	0.9 ± 0.2	1.0 ± 0.2	1.0 ± 0.2	
**F value, p (by age category)**	1.228,0.299			
**Dietary diversity scores**				
6 – 8.99 mos	1.0 ± 0.8	0.7 ± 0.7	0.9 ± 0.8	0.145,0.703
9 – 11.99 mos	0.7 ± 0.7	0.5 ± 0.7	0.6 ± 0.7	
12 – 17.99 mos	0.8 ± 0.8	0.8 ± 0.8	0.8 ± 0.8	
18 – 24 mos	0.8 ± 0.8	0.9 ± 0.8	0.9 ± 0.8	
**F value, p (by age category)**	3.035,0.029			
**Food group frequency scores**				
6 – 8.99 mos	1.5 ± 0.6	1.3 ± 0.8	1.4 ± 0.7	0.275,0.600
9 – 11.99 mos	1.8 ± 0.5	1.6 ± 0.7	1.7 ± 0.6	
12 – 17.99 mos	1.7 ± 0.5	1.8 ± 0.5	1.7 ± 0.5	
18 – 24 mos	1.8 ± 0.4	1.9 ± 0.3	1.9 ± 0.4	
**F value, p (by age category)**	3.796,0.010			
**Feeding frequency scores**				
6 – 8.99 mos	1.6 ± 0.7	1.1 ± 0.9	1.3 ± 0.8	0.059,0.807
9 – 11.99 mos	0.9 ± 0.9	0.8 ± 1.0	0.9 ± 0.9	
12 – 17.99 mos	1.1 ± 1.1	1.3 ± 1.3	1.2 ± 1.2	
18 – 24 mos	1.5 ± 1.1	1.4 ± 1.2	1.5 ± 1.1	
**F value, p (by age category)**	1.267,0.285			

Mean values ± standard deviation.

Mean scores were approximately half the maximum possible score of 2, regardless of age category and sex. The percentage of children who were fed foods from the seven food groups were: grains, roots and tubers - 91.7%; legumes and nuts - 59.6%; milk/ milk products - 58.1%; flesh foods - 2.7%; eggs - 5.4%, vitamin A rich fruits and vegetables - 8.7% and other fruits and vegetables - 19.7%. DDS scores of children between 9 and 17.99 months had significantly lower scores than the 6 to 8.99 month old children, with the lowest mean scores being observed for the 9 to 11.99 month age group.

In contrast to DDS scores, FGFS scores increased with age. The mean FGFS score was significantly higher among children >12 months of age (1.77 ± 0.5) compared to children <12 months of age (1.53 ± 0.6; F value = 18.749, p = 0.000). Also, the mean FGFS score was significantly higher for non-breastfed children (1.85 ± 0.5) compared to breastfed children (1.64 ± 0.6; F value = 7.001, p = 0.008).Gender was not significantly associated with FGFS. Scores for FGFS were better than DD scores since they were more than half the maximum possible score of 2.

For FFS, the maximum possible was 2 for children <12 months old and 3 for children >12 months of age. Children <12 months old were better off as the difference between the maximum score and mean scores was 0.90-1.20, whereas the older children were worse off, since the difference between their mean scores and the maximum score of 3 was 1.50-1.90.

The mean FFS was higher among children >12 months of age (1.29 ± 1.2) compared to children <12 months of age (1.11 ± 0.9), however there was no significant difference between the two age categories. The mean scores for 9 – 11.99 month old children was the lowest among the four age categories. Further, mean FFS was significantly higher among non-breastfed (1.63 ± 1.1) compared to breastfed children (1.14 ± 1.1; F = 11.569, p = 0.001).

The percent distribution of children for the different ICFI components by age category and gender was examined (Table 4). Scores for breastfeeding, did not differ significantly by age because almost all mothers of children less than 12 months of age and approximately three-fourth mothers of children above 12 months breastfed their children regardless of gender of the child. More than 95% of mothers did not use a bottle for feeding, irrespective of gender and age group.

**Table 4:** **Percent distribution of ICFI components by age and gender**

**ICFI components**	**6 – 8.99 mos**		**9 – 11.99 mos**		**12 – 17.99 mos**		**18 – 24 mos**		**All children**		
	**Male**	**Female**	**Male**	**Female**	**Male**	**Female**	**Male**	**Female**	**Male**	**Female**	**Total**
**Breastfeeding**											
Yes	93.8	98.0	93.5	88.6	85.9	81.7	68.8	78.6	86.7	86.3	86.6
**χ** ^**2**^ **, p (by age)**	2.224,0.329		1.567,0.457		0.612,0.434		0.737,0.391		0.022,0.989		
**Bottle-feeding**											
No	95.9	98.0	95.7	97.7	97.8	98.1	93.8	96.4	96.3	97.8	97.1
**χ** ^**2**^ **, p (by age)**	0.386,0.534		0.301,0.584		0.015,0.901		0.226,0.635		0.829,0.363		
**Dietary diversity score**											
Low (0)	26.5	49.0	50.0	61.4	45.7	42.3	43.8	32.1	42.0	46.3	44.2
Medium (1)	42.9	35.3	34.8	27.3	32.6	34.6	28.1	42.9	34.7	34.4	34.5
High (2)	30.6	15.7	15.2	11.4	21.7				23.3	19.4	21.3
**χ** ^**2**^ **, p (by age)**	6.113,0.047		1.181,0.554		0.222,0.895		1.506,0.471		1.257,0.534		
**Food group frequency score**											
Low (0)	8.2	17.6	4.3	11.4	4.3	4.8	3.1	0.0	5.0	8.4	6.7
Medium (1)	32.7	37.3	15.2	18.2	19.6	15.4	9.4	7.1	20.1	19.8	20.0
High (2)	59.2	45.1	80.4	70.5	76.1	79.8	87.5	92.9	74.9	71.8	73.3
**χ** ^**2**^ **, p (by age)**	2.834,0.242		1.838,0.399		0.601,0.740		1.012,0.603		2.005,0.367		
**Feeding frequency score**											
Low (0)	12.2	31.4	45.7	52.3	39.1	37.5	25.0	32.1	32.4	38.3	35.4
Medium (1)	18.4	31.4	15.2	20.5	23.9	20.2	25.0	17.9	21.0	22.5	21.7
High (2/3)*	69.3	37.3	39.1	27.2	37.0	42.3	50.0	50.0	46.6	39.2	42.8
**χ** ^**2**^ **, p (by age)**	10.882,0.012		4.584,0.205		5.186,0.159		0.774,0.856		11.435,0.010		

*2 for 6 to 11.99 month old age group and 3 for 12 to 24 month age group. Absolute numbers (%).

About one-fifth of all children had high DDS. The mean DDS of children aged <12 months (0.72 ± 0.8) was low compared to children aged >12 months (0.81 ± 0.8), however the difference was not statistically significant. The mean score was significantly higher for non-breastfed (0.95 ± 0.7) than breastfed children (0.74 ± 0.8, F value = 4.230, p = 0.040). DDS was examined in relation to gender but no significant association was observed, although among children <12 months, females tended to be worse off as 49% of 6 – 8.99 month old girls and 61.4% of the 9 – 11.99 month old had low DDS scores compared to 26.5% and 50% of boys in the same age categories. A little less than one-fourth of male children (<12 months) had a high DDS compared to only 13.7% females. When the <12 month age group was sub-divided further into 6 – 8.99 months and 9 – 11.99 months, girls in the 6 – 8.99 month age group were worse off as only 15.7% had high DDS compared to almost twice the percentage of boys (30.6%). Among the 9 – 11.99 month old children, a slightly lower percentage of girls had high DDS than did boys. In the older age group (>12 months), there was no significant difference between the two sexes.

About three fourths of the children had high FGFS scores (score of 2) and about one – fifth had medium scores (score of 1). The percentage of children with high FGFS scores increased with age (Table 4), with the lowest percentage being in the 6 – 8.99 months age group. Comparison between age groups within gender showed that there was no significant difference in the distribution for boys (χ^2^ = 10.051, p = 0.123). However, girls below 12 months of age were worse off compared to girls above 12 months of age (χ^2^ = 28.550, p = 0.000).

A little more than half of males (54.8%) <12 months of age had a higher feeding frequency score compared to one-third of females (32.7%) in the same age category. However, among children aged >12 months, there was no significant association between feeding frequency score (FFS) and gender of the child. When the four age categories were compared, the gender difference became more pronounced for the 6 – 8.99 month age group (Table 4). A much higher percentage of males (69.3%) had high FFS compared to 37.3% of females. Further, the percentage of children with high FFS was lowest in the 9 – 11.99 month age group followed by the 12 – 17.99 month old age group. Even among children above 18 months of age, about one – fourth to one – third of the children had low FFS, although it was lower compared to children aged 9 – 17.99 months indicating that a substantial proportion of the children were under – fed.

Step – wise regression analysis was done using the scores of the five individual components as independent variables and total ICFI score as the dependent variable. The variables were entered in the model in following order: DDS, FFS, breastfeeding score, FGFS and bottle feeding score. Overall the model was significant (F value = 2.055E + 16, p = 0.000). All the five components were found to be significant, with DDS (Beta = 0.405, p = 0.000) and FFS (Beta = 0.561, p = 0.000) contributing the most to the total ICFI score followed by breastfeeding score (Beta = 0.354, p = 0.000), FGFS (Beta = 0.311, p = 0.000) and bottle feeding score (Beta = 0.088, p = 0.000).

### Association of ICFI with nutritional status of children

The mean z scores by age categories and ICFI categories were examined (Table 5). There was no significant difference between mean z scores for WLZ, WAZ, LAZ, BAZ and MUACZ in the low, medium and high categories of ICFI.

**Table 5:** **Mean Z scores and distribution of children (%) with Z scores < −2SD by ICFI categories**

**Age category**	**ICFI category**	**n**	**WLZ**	**LAZ**	**WAZ**	**BAZ**	**MUACZ**
6 – 8.99 mos (n = 100)	Low (0–5)	37	−1.0 ± 2.6	−0.6 ± 2.5	−1.3 ± 1.7	−1.3 ± 2.4	−0.3 ± 1.2
	Medium (6–7)	25	−0.9 ± 2.5	−0.7 ± 2.1	−1.2 ± 1.8	−1.1 ± 2.5	−0.7 ± 0.9
	High (8–9)	37	−0.6 ± 2.1	−0.4 ± 2.4	−0.9 ± 1.5	−0.8 ± 2.0	−0.4 ± 1.3
9 – 11.99 mos (n = 90)	Low (0–5)	45	−1.0 ± 2.4	−1.1 ± 2.3	−1.5 ± 1.4	−1.1 ± 2.3	−0.3 ± 0.9
	Medium (6–7)	23	−0.7 ± 2.9	−1.6 ± 2.2	−1.5 ± 1.7	−0.7 ± 2.9	−0.8 ± 1.1
	High (8–9)	22	−1.4 ± 1.9	−2.2 ± 1.9	−2.2 ± 1.5	−1.4 ± 1.9	−0.5 ± 1.3
12 – 17.99 mos (n = 196)	Low (0–5)	100	−0.1 ± 2.0	−3.1 ± 2.3	−1.8 ± 1.3	0.2 ± 1.9	−0.8 ± 1.2
	Medium (6–7)	53	0.2 ± 2.5	−2.1 ± 2.2	−1.2 ± 1.4	0.1 ± 2.7	−0.4 ± 1.3
	High (8–9)	43	−0.9 ± 2.0	−2.5 ± 2.2	−2.0 ± 1.3	−0.7 ± 2.1	−0.5 ± 1.3
18 – 24 mos (n = 60)	Low (0–5)	25	−0.2 ± 1.7	−4.7 ± 2.0	−2.7 ± 1.2	0.5 ± 1.7	−1.4 ± 1.4
	Medium (6–7)	18	−0.8 ± 2.8	−4.3 ± 2.5	−2.9 ± 1.7	−0.2 ± 3.0	−1.5 ± 0.7
	High (8–9)	16	−0.5 ± 2.2	−4.3 ± 1.8	−2.7 ± 1.2	0.2 ± 2.4	−1.1 ± 1.1
**F value, p (by ICFI categories)**			0.502,0.605	0.245,0.783	0.681,0.506	0.447,0.640	0.934,0.394
**Percent children with Z scores < −2 SD**							
6-8.99 mos	-	100	29.4	9.6	15.1	32.8	6.0
9-11.99 mos	-	90	26.1	14.8	21.5	28.6	5.5
12-17.99 mos	-	196	36.1	52.8	39.8	32.8	15.3
18-24 mos	-	59	8.4	22.7	23.7	5.9	31.6

Mean values ± standard deviation.

Multivariate regression analysis was carried out to determine whether selected maternal and child characteristics were associated with ICFI. The variables entered in the equation were maternal age, BMI, education, family income, child’s age, sex and ordinal position. The model was significant (R^2^ (R^2^ Adj) = 0.046(0.028); F value = 2.617, p = 0.008). Among these maternal age was positively and significantly associated (Beta = 0.153, p = 0.006), whereas child’s age was negatively and significantly associated (Beta = −0.015, p = 0.026) with ICFI score.

Further, multivariate regression analysis was performed to determine if there was significant association between ICFI and nutritional status after controlling for other confounding variables namely - child characteristics (age, gender and ordinal position), maternal characteristics (age, BMI and education) and household characteristics (per capita income). In the multivariate model (Table 6), ICFI was significantly associated with LAZ and BAZ scores, but not with WLZ, WAZ and MUACZ. Other variables that had significant impact on nutritional status were: BMI of mother and mothers completing secondary schooling for WLZ and BAZ, age of the mother for MUACZ, gender of the child for WAZ and LAZ. Age of the child had the most significant impact on all indicators of nutritional status except WLZ.

**Table 6:** **Multivariate regression analysis of determinants of nutritional status**

**Parameters**	**WLZ**		**WAZ**		**LAZ**		**BAZ**		**MUACZ**	
	**Beta**	**Sig**	**Beta**	**Sig**	**Beta**	**Sig**	**Beta**	**Sig**	**Beta**	**Sig**
**Maternal characteristics**										
Age (yrs)	0.050	0.368	0.093	0.083	1.001	0.317	0.684	0.494	3.008	0.003
Per capita income (Rs)	−0.038	0.448	−0.042	0.384	0.075	0.940	−0.976	0.330	0.122	0.903
BMI	0.107	0.029	0.068	0.148	−1.287	0.199	2.485	0.013	0.354	0.724
≤SSC	0.157	0.003	0.085	0.092	−1.888	0.060	3.237	0.001	0.041	0.967
≥HSC	0.086	0.100	0.047	0.352	−1.120	0.263	1.712	0.088	−0.656	0.512
**Child characteristics**										
Gender	0.005	0.910	0.091	0.046	2.610	0.009	−0.163	0.871	1.198	0.231
Age (months)	0.052	0.273	−0.303	0.000	−11.344	0.000	3.910	0.000	−4.397	0.000
Ordinal position	−0.024	0.679	−0.066	0.230	−0.770	0.442	−0.519	0.604	−1.595	0.111
**ICFI score**	−0.092	0.056	−0.010	0.827	2.034	0.043	−2.012	0.045	0.510	0.610
**R** ^**2**^ **(R** ^**2**^ **Adj)**	0.051(0.032)		0.115(0.096)		0.263(0.247)		0.093(0.074)		0.066(0.047)	
	F value = 2.622,		F value = 6.266,		F value = 17.258,		F value = 4.948,		F value = 3.415,	
	p = 0.006		p = 0.000		p = 0.000		p = 0.000		p = 0.000	

Sensitivity and specificity of ICFI were also calculated. The sensitivity and specificity of the indicators of nutritional status are presented in Table 7. The sensitivity i.e. ability of the index to correctly identify children as undernourished ranged from 16 to 54% and specificity (ability of the index to correctly identify children as normal) ranged from 51 to 89%. However, sensitivity was highest for LAZ followed by WAZ but was very low for WLZ, BAZ and MUACZ. Specificity was highest for MUACZ followed by WLZ and BAZ.

**Table 7:** **Sensitivity and Specificity of Total ICFI Score with Indicators of Nutritional Status**

	**Sensitivity** ^*^ **(95% CI)**	**Specificity** ^**#**^ **(95% CI)**	**P value**
WLZ	0.236(0.191,0.282)	0.705(0.666,0.745)	0.164
LAZ	0.545(0.494,0.597)	0.515(0.469,0.560)	0.218
WAZ	0.435(0.384,0.486)	0.599(0.554,0.644)	0.501
BAZ	0.230(0.186,0.276)	0.700 (0.662,0.741)	0.108
MUACZ	0.163 (0.127,0.198)	0.890 (0.859,0.920)	0.126

^*^Ability of the index to correctly identify children as undernourished.

^#^Ability of the index to correctly identify children as normal.

## Discussion

The present study provides data on selected quantitative aspects of complementary feeding practices in urban slum settings in Mumbai, India, by using the ICFI developed by Arimond and Ruel, and determining its association with five indicators of nutritional status [19]. A significant association was found between ICFI and LAZ and BAZ in this study sample.

The prevalence of undernutrition specifically stunting followed by underweight and wasting was high in the present study, which could be partly attributed to poor complementary feeding practices, lack of knowledge among mothers/caregivers, poor hygiene and low socio-economic status [20-22].

In the present study, the mean ICFI score was 5.9 out of a maximum possible score of 9 indicating that some of the child feeding practices that are assessed in the index were inappropriate. The five components of the ICFI are breastfeeding, bottle feeding, dietary diversity, food group frequency and feeding frequency. While there was not much difference between age groups, in the percentage of children who were breastfed or bottle-fed, at least half of the 9 – 11.99 month age group had low DDS and about half had low scores for feeding frequency. Out of the maximum possible ICFI score of 9, almost half i.e. 4 to 5 score points are contributed together by DDS and FFS. This trend was also observed for older children above 12 months of age, although a higher percentage of 9 – 11.99 month old children had lower scores than the older age group. The relatively low scores for these two individual components indicates that feeding practices after 9 months require considerable attention and that between 9 to 24 months of age, the 9 to 11.99 month period in infancy perhaps needs to be closely focused on for interventions aimed at behaviour change and improving child nutrition. Garg and Chadha studied rural children in a narrow age range of 6 – 12 months in Ghaziabad district, Uttar Pradesh, North India [6]. However their index included timely initiation of complementary feeding which is not included in the ICFI index used in this study.

In the present study, ICFI was found to be significantly associated with LAZ but not with WAZ or WLZ. Other reports in the literature indicate that LAZ was significantly associated with the child feeding index in several countries including Bolivia, Colombia, Guatemala, Nicaragua, Peru, Ethiopia and India [6,12,17]. In Burkina Faso, Sawadago et al. used a modified ICFI and observed a significant relationship with LAZ of children aged 6–36 months of age [13]. Garg and Chadha also found significant association between a complementary feeding index that they developed and LAZ in rural Indian children [6]. In Bangladesh, Khatoon et al. found a significant relationship between ICFI and LAZ, especially among children aged 12–23 months of age. Similarly, Bork et al. reported significant association between ICFI and LAZ among Senegalese children below 12 months of age [15,16]. Recently, Ma et al. reported positive association between ICFI and LAZ and WAZ among Chinese children of 18 months of age [7]. Olabiyi [Complementary Feeding Practices and Nutritional Status of Children 6–24 Months in Abeokuta South Local Government Area, Ogun state, Nigeria, Unpublished Project Report, 2012] also reported a significant association between complementary feeding practices of the caregivers and WAZ. However, this finding does not conform with the results reported by Ntab et al. in Senegal, Moursi et al. in Madagascar [14,23]. These investigators reported that the index was unable to report impact on nutritional status in their multivariate model.

Black et al. reported that even with optimum breastfeeding, children will become stunted if they do not receive an adequate quantity and quality of complementary foods after six months of age [1]. Our findings are in line with the report by Black et al. [1]; as DDS and FFS scores for these children were relatively poor and both together contributed to about half of the total score. Inappropriate feeding practices that provide inadequate amounts of important macro and micronutrients over a long period will result in compromised growth that will reflected by poorer LAZ scores. Malik and Mazhar [24] reported that the odds ratio of children being malnourished was 2.54 times for children who received complementary foods after one year of age compared to children who were given complementary foods before the age of four months. In the present study, stunting was higher among older children than children <12 months of age [5,25]. This needs attention since stunting is associated not only with poor physical growth but also affects cognitive abilities that are irreversible after 2 years of age [26].

The association between ICFI and nutritional status in the multivariate model in the present study showed that even after controlling for selected maternal, child and household characteristics, infant and child feeding practices were important determinants for LAZ and BAZ. These were poor diversity of complementary foods and poor feeding frequency of the complementary foods. Further, age and gender of child, maternal education and BMI of mother were important factors influencing nutritional status which was also demonstrated by Arimond and Ruel, Armar-Klemesu et al. Dewey et al. and Srivastava and Sandhu [5,21,27,28]. Also, multivariate regression analysis indicated that mother’s age and education are important factors. In developing country settings, poor maternal education level and young age reflect women’s status and their care capacity.

The findings of the present study confirm the findings that ICFI may be able to reflect chronic malnutrition among young children, however, the sensitivity is not very high [5-7]. This index reflected the quality and quantity of the complementary foods fed to the children in urban slum setting in terms of the food frequency and dietary diversity scores and indicated that poor quality reflected by the DD scores and low frequency of complementary foods were major factors determining the nutritional status of the child [5,6,15]. Thus, improvement in dietary diversity, quality and frequency of feeding complementary foods needs to be addressed through appropriate interventions in order to improve feeding practices and nutritional status of children under two years of age.

The strengths of the present study are that it is perhaps one of the first to use the ICFI for children between 6 to 24 months in urban slum setting. We are one of the few who have examined sensitivity and specificity of ICFI, however a larger sample size may be worthwhile to come to a conclusion, and further longitudinal examination of the data is required to examine time-trend relationships. The limitation of the study is that in the index, age of initiation of complementary foods was not considered. In the present study, 9 to 12 month identified as the most vulnerable age period as 8% children from the same age group did not receive complementary foods until 9 months of age, but this needs to be confirmed with a larger sample size. The ICFI index needs to be validated in the Indian sample. Only a small variance was explained by the maternal and child characteristics (R^2^ Adj: 0.028) therefore, other factors needs investigation which may determine feeding practices such as influence of extended family members e.g. mother – in – law, maternal self – efficacy, parity, socio – economic status, standard living index, etc.

## Conclusions

The results of the present study confirmed that the ICFI index can be used to collect information on various components of young child feeding practices. It can be used in public-health programmes for addressing the issue of complementary feeding as a whole and also for monitoring the change in feeding practices. Further, it could be used to determine the influence of complementary feeding practices on nutritional status of children.

The study points out the need for intensive nutrition education and improving women’s status in terms of education and delaying age of marriage in order to improve infant and young child nutritional status.

## Abbreviations

ICFI: Infant and Child Feeding Index
IYCF: Infant and young child feeding practices
WLZ: Weight-for-length z score
WAZ: Weight-for-age z score
LAZ: Length-for-age z score
BAZ: Body mass index- for- age z score
MUACZ: Mid-upper arm circumference-for-age z score
MUAC: Mid-upper arm circumference
BMI: Body mass index
DDS: Dietary diversity score
FGFS: Food group frequency score
FFS: Feeding frequency score
